# Towards Fingerprint Spoofing Detection in the Terahertz Range

**DOI:** 10.3390/s20123379

**Published:** 2020-06-15

**Authors:** Norbert Pałka, Marcin Kowalski

**Affiliations:** Institute of Optoelectronics, Military University of Technology, 00-908 Warsaw, Poland; marcin.kowalski@wat.edu.pl

**Keywords:** fingerprint spoof detection, terahertz radiation, time domain spectroscopy, neural networks

## Abstract

Spoofing attacks using imitations of fingerprints of legal users constitute a serious threat. In this study, a terahertz time domain spectroscopy (TDS) setup in a reflection configuration was used for the non-intrusive detection of fingerprint spoofing. Herein, the skin structure of the finger pad is described with a focus on the outermost stratum corneum. We identified and characterized five representative spoofing materials and prepared thin and thick finger imitations. The complex refractive index of the materials was determined in TDS in the transmission configuration. For dataset collection, we selected a group of 16 adults of various ages and genders. The reflection results were analyzed both in the time (reflected signal) and frequency (reflectivity) domains. The measured signals were positively verified with the theoretical calculations. The signals corresponding to samples differ from the finger-related signals, which facilitates spoofing detection. Thanks to deconvolution, we provide a basic explanation of the observed phenomena. We propose two spoofing detection methods, predefined time–frequency features and deep learning based. The methods achieved high true detection rates of 87.9% and 98.8%. Our results show that the terahertz technology can be successfully applied for spoofing detection with high detection probability.

## 1. Introduction

The fingerprint is a unique and secure biometric trait, which has been widely used in forensic investigations, financial transactions, security access control systems, and for border control verification [1,2,3,4]. At the present time, many fingerprint scanners are widely available, ensuring their diversity and universality of applications. Many countries store fingerprints of citizens in electronic passports and IDs and register biometric data in databases with millions of records (e.g., US-origin automated biometric identification system, EU-origin visa information system). Although today’s state-of-the-art fingerprint systems are very fast and provide high recognition accuracy, they can be exposed to attacks at the sensor level, replay attacks on the data communication stream and attacks on the databases [1,2,3]. Presentation attacks using imitations made of silicone, gelatin or Play-Doh with fingerprints of legal users are quite common. Therefore, fingerprint scanners should detect spoofing attempts with high probability to ensure that only biometric data from a live person is submitted for registration and authentication.

Several liveness detection solutions have been proposed to cope with spoofing attacks. The software-based solutions use sophisticated analysis of images captured by standard readers to detect life signs in acquired fingerprints. These include specific skin deformation during touching the scanner surface, ridge and valley texture, perspiration, surface coarseness and presence of natural pores or unnatural features [1,2,3,5,6]. Software-based anti-spoofing methods extract salient features from the fingerprint images to separate live and spoofed samples. A variety of methods are used to extract features from fingerprint images, including hand-crafted features (Weber local binary descriptor [7], scale-invariant feature transform (SIFT) [8]). Current state-of-the-art solutions use convolutional neural networks (CNN) [9,10,11,12,13] to learn feature representation of fingerprints.

Current research in the field of algorithms aims to address the generalization aspect of fingerprint spoofing. It has been shown that the selection of spoof materials used during the training process influences the performance against unknown spoofs [14,15]. Chugh and Jain [16] proposed a style-transfer-based method to improve the cross-material and cross-sensor generalization performance of fingerprint spoof detectors. Chugh et al. [11] proposed a CNN-based method trained on patches around minutiae points which achieved impressive average accuracy of 98.61%. However, the cross-material validation showed significant decrease of performance. Zhang et al. proposed Slim-ResCNN: a deep residual convolutional neural network for fingerprint liveness detection [17], a new CNN architecture with residual blocks that achieved an overall accuracy of 95.25%. Chugh and Jain [18] proposed a style-transfer-based wrapper, called the Universal Material Generator (UMG), to improve generalization performance of any fingerprint spoof detector. The proposed approach was shown to improve performance of the Fingerprint SpoofBuster [19] and Slim-ResCNN [17] methods to a true detection rate (TDR) of 91.78% and 90.63%, respectively, at a false detection rate (FDR) of 0.2%.

Hardware-based detection methods use additional sensors to measure life signs, such as finger temperature, pulse oximetry, blood pressure and odor [1,2,3]. This group also exploits devices operating in visible and near-infrared ranges, such as optical coherence tomography [20] and multispectral sensors [21]. The last solution is based on multiple wavelengths of light with different polarizations to acquire relevant information about the finger pad properties [22]. Skin is a highly inhomogeneous and complex layered structure which renders it hard to mimic, especially when optical waves are used for examination. On the other hand, the main problem in the liveness detection is the wide variety of living fingers [23]. Engelsma proposed an open-source fingerprint reader, called RaspiReader, that uses two cameras to provide direct-view and frustrated total internal reflection (FTIR) images for spoof detection [24]. This method [24] reached impressive spoof detection performance with a TDR of 100% in a cross-material scenario for different materials, except those that were highly transparent. Qualcomm proposed ultrasound-based in-display fingerprint readers for smartphones [25] which utilize acoustic response characteristics for spoof detection.

Spoofing detection performance of different fingerprint algorithms and systems has been studied and compared during Liveness detection competition (LivDet) [26,27,28,29] since 2009. This competition created a benchmark for measuring liveness detection algorithms. The competitions aim to allow researchers to test their algorithms and systems on publicly available datasets, obtained and collected with the most updated techniques. In 2015, the LivDet [28] competition introduced cross-material validation. In 2019, LivDet [26] focused on investigation of how the integration of a liveness detection system with a fingerprint authentication system may impact the performance. Therefore, the spoofing detection algorithms were evaluated together with sensors.

In the past decade, terahertz (THz) radiation within the range of 0.1–3 THz has been used in a growing number of applications, including explosive detections [30], security screening [31,32], nondestructive evaluation [33], telecommunication [34] and biomedical investigations [35,36,37,38,39,40,41,42]. Terahertz radiation penetrates a variety of nonpolar non-conducting materials and is inherently safe for living tissues and DNA, because it is not ionizing thanks to low photon energy. Due to high attenuation of water, penetration of living tissues is limited to a few millimeters at lower frequencies of the THz band. THz radiation, as non-invasive and highly sensitive to water, has a potential for in vivo medical imaging and diagnosis, including evaluation of early stage deterioration of diabetic patients’ feet [39], corneal tissue hydration sensing [42], detection of cancer tissues [41] and identification of skin cancer [42]. For more than 10 years, human skin has been extensively studied to better understand the interaction of THz radiation with complex tissue structure and pathologic changes. Due to high attenuation of water, in vivo terahertz measurement of human skin can only be carried out in reflection configuration.

Fingerprint spoofing detection in the THz domain has not been yet widely explored. In [43] a THz microscopy technique to acquire images of the human skin is proposed. The proposed system detects the sweat ducts along with other surface skin traits. Detection of sweat ducts may be used as spoofing indicator. However, authors of [43] have not evaluated the proposed method against presentation attacks.

Among many terahertz reflection methods, time domain spectroscopy (TDS) seems to be particularly useful in research with live samples because it can provide fast spectroscopic information about examined samples. TDS generates and detects about 1-ps-long pulses of electromagnetic radiation in a synchronized way. These pulses have a broad spectrum, usually in the range of 0.1–3 THz. Basic components of this setup are a femtosecond laser, two photoconductive antennas and a delay line. The detailed description of the TDS technique can be found in [44]. The TDS technique may provide information on the inner structure of various materials, in addition to looking into nonmetallic layered structures thus may be useful for detection of additional layers attached to the fingerprint.

In this study, we present experimental results of the TDS technique for detection of spoofed fingerprints. The contributions are summarized as follows:

- Introduction of hardware-based fingerprint anti-spoofing method using the THz TDS technique;
- Investigations of fingerprint presentation attack detection in terahertz time domain spectroscopy;
- THz time domain characterization of various presentation attack instruments (PAIs);
- Two presentation attack detection methods based on:
  (a) time–frequency feature analysis;
  (b) a deep learning classifier applied to reflectance spectra with a second criterion analysis based on reflected signals in the time domain.

The organization of the study is as follows: Section 2 briefly presents the finger pad skin structure. Section 3 describes finger imitations prepared for research and characterizes their terahertz properties. The experimental setup is shown in Section 4. Section 5 describes analysis of experimental results for the imitations, while Section 6 does so for real finger pads. The spoofing detection methods with results of different validation schemes are presented in Section 7.

## 2. Finger Pad Skin Structure

**Figure 1:**
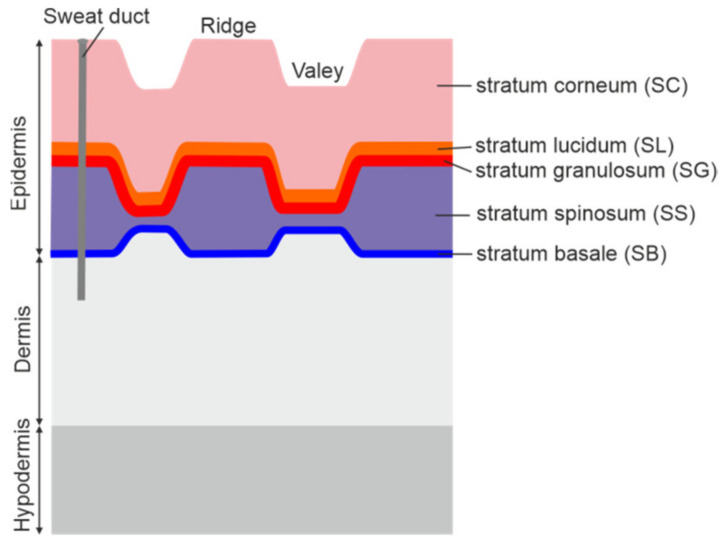
Simplified scheme of friction ridge skin. Based on [46].

The human skin consists of three main layers: the epidermis, the dermis and the hypodermis as presented in Figure 1. The epidermis constitutes the top layer of the skin and includes five layers. Starting from the outside, these are: stratum corneum (SC), stratum lucidum (SL), stratum granulosum (SG), stratum spinosum (SS) and stratum basale (SB) [45].

The outermost stratum corneum consists of about 15–20 layers of dead cells which form a barrier to protect underlying tissue from infection, dehydration, chemicals and mechanical stress. The skin of fingers, called friction ridge skin, features the presence of raised ridges; its epidermis is thicker and more complex [46]. The thickness of the stratum corneum ranges roughly from 0.2 to 0.6 mm [47] and depends on many factors. In the THz investigations, due to high attenuation of the skin and resultant low penetration depth, the epidermis affects the reflected THz signal the most.

The width of fingerprint ridges is 0.1–0.4 mm and the width of valleys is 0.1–0.2 mm, while the depth of valleys is 0.1–0.2 mm [48]. However, when the ridges come into contact with the window surface, due to the pressure applied, the valley depth is reduced to about 50 µm [49].

## 3. Characterization of Samples

The considered terahertz technique was used to distinguish skin of real finger pads from their imitations (overlays). Thus, we prepared flat cuboid samples with a working area of about 1 × 1 cm^2^ [5]. We selected easily available ingredients and materials to prepare spoofing samples and to show the overall potential of the technique, rather than testing all possible materials. After the analysis, we created thin and thick spoofing samples from five representative materials (Table 1). The thickness range of the samples resulted from realistic thicknesses of the overlays that could be made at home and attached to fingers.

**Figure 2:**
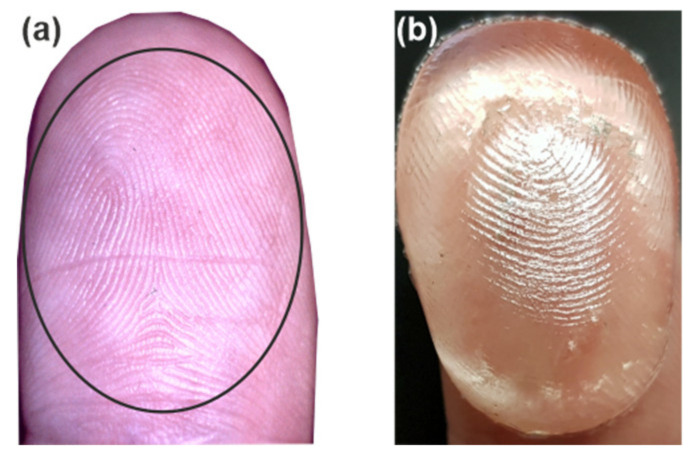
Real fingerprint (**a**) imprinted on gelatin-sample G3 (**b**). The ellipse defines the finger pad, which was analyzed by THz radiation.

Gelatin, due to its high water content, is similar to skin. Thus, we prepared a mold and cast a gelatin finger overlay (sample G3) with a copy of real fingerprints (Figure 2). At the beginning of our experiments, we proved experimentally that usage of samples with or without imprinted fingerprints did not influence the reflected THz signal. We think that when the fingerprint pattern comes into contact with the window surface, the applied pressure decreases the valleys and ridges so far that they are invisible to the THz waves. The size of the fingerprint structures was too small to interact with the comparatively low spatial-resolution THz system with a beam size of a few millimeters.

**Figure 3:**
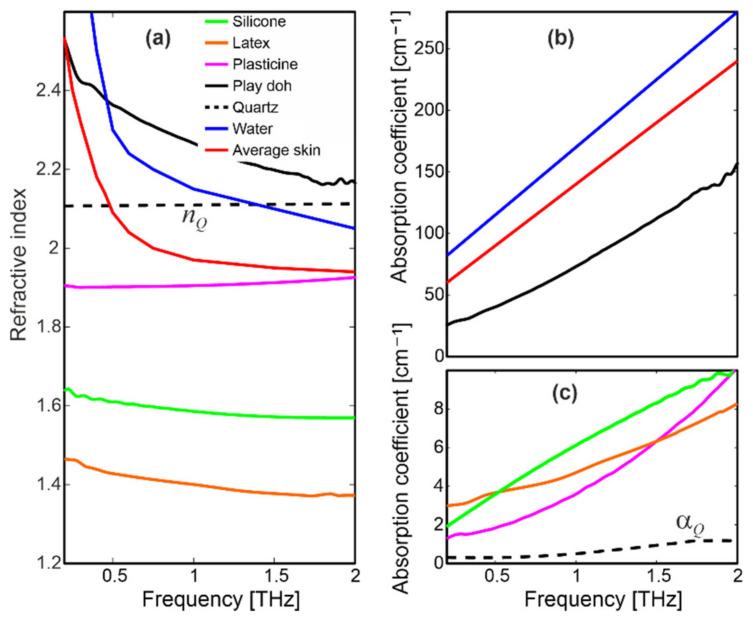
(**a**) Refractive index and (**b**,**c**) absorption coefficient of the studied samples and materials.

A TDS spectrometer Spectra 3000 from TeraView in a transmission configuration [50] was used to determine the complex refractive index (*N* = *n* + i*κ*) of the studied samples (Figure 3); *n* is the refractive index while *κ* is the extinction coefficient, usually represented by the absorption coefficient (*α4πνκ*/*c*), due to its intuitive interpretation; ν is the frequency of the radiation and *c* is the speed of light.

Silicone, latex and plasticine constituted materials Group I. They are quite transparent for THz radiation (*α* < 10 cm^−1^), while their refractive indices are roughly in the range of 1.4–1.9. Both *n* and *α* slightly change as a function of frequency.

On the other hand, Play-Doh features a high and monotonically increasing attenuation (70 cm^−1^ @ 1 THz), while its refractive index profile was relatively high (2.5 @ 0.1 THz) and decreased to about 2.2 for higher frequencies, which suggests this material has strong dispersion properties. The gelatin sample could not be measured in the transmission setup, because it consists of highly attenuating water. Therefore, instead of gelatin we present *n* and *α* characteristics of water based on the literature [51]. The curves were similar to the Play-Doh sample, but the attenuation was much higher (about 170 cm^−1^ @ 1 THz). The water-based samples constituted materials Group II.

Terahertz characteristics of skin presented in the literature [51] differ because they depend on many factors, including place of measurements and state and age of the person, as well as measurement setup and conditions. Based on comparisons presented in [51], we plotted an average complex refractive index of human skin, which should be treated as a general trend.

Moreover, we plotted the complex refractive index of a quartz window (*N_Q_*), which was part of the measurement system. This material features an almost constant refractive index value *n_Q_* = 2.10, while its absorption coefficient was very low (*α_Q_* < 1 cm^−1^). Similar data are shown in literature [52].

For testing, we selected a group of 16 adults, who differed in age (25–80 years) and gender. All subjects gave their informed consent for inclusion before they participated in the study. The study was conducted in accordance with the Declaration of Helsinki, and the protocol was approved on 20/01/2020 by the Ethics Committee of Institute of Optoelectronics, Military University of Technology. The pads of the index, middle, and ring fingers of the right hand were scanned. During the measurements, the skin was cleaned and free of creams and other cosmetics.

## 4. Experimental Setup

**Figure 4:**
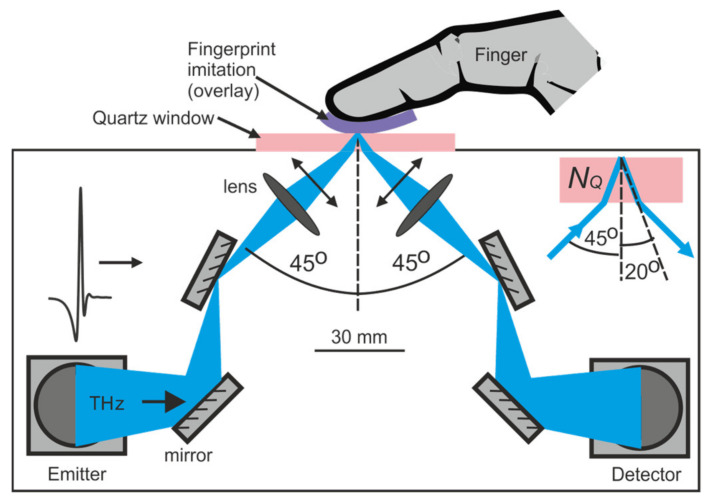
Experimental setup.

The TDS system TPS 3000 (TeraView) [50] was used to measure time domain signals reflected from the samples (Figure 4). The THz pulse generated in the emitter was reflected by the mirrors and focused by the lens on the upper surface of the quartz window, where the fingers and overlays were placed. Quartz is commonly used in similar terahertz experiments with skin [35], because it is hard, durable and transparent for both terahertz and optical waves. A single-crystal α-quartz wafer with thickness of 2 mm and diameter of 30 mm was z-cut, which provided a lack of birefringence. After interacting with the samples, the signal was guided back to the receiver.

The p-polarized (transverse magnetic) THz beam was incident on the bottom surface of the window at the angle *θ*_0_ = 45°, and was refracted and hit the upper surface at the angle *θ*_1_ = 20°, which was calculated by the Snell law (*n_Q_* = 2.10). The frequency-dependent beam diameter was estimated to be about 4–3 mm at lower frequencies (0.1–0.5 THz) and 2–1 mm at higher frequencies (1–2 THz). This beam size was lower than the sample sizes and therefore ensured uniform interaction with the samples. The THz chamber was purged with dried air to suppress the influence of water vapor.

**Figure 5:**
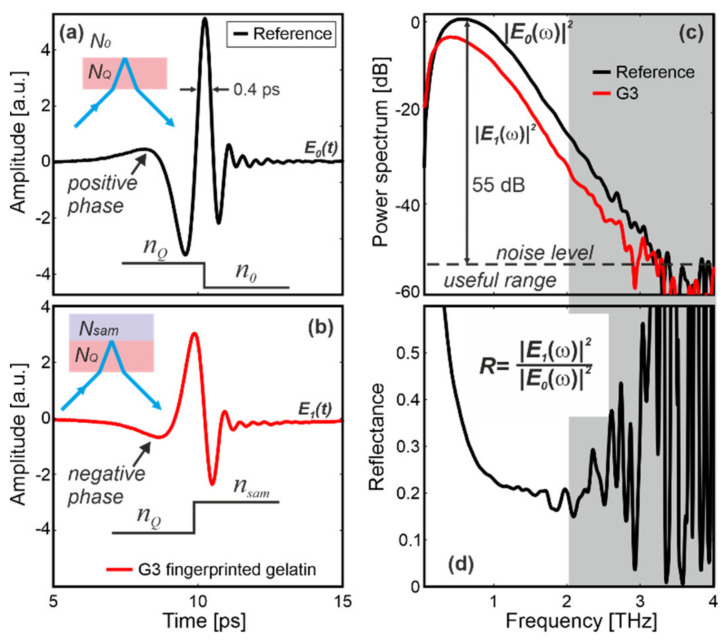
(**a**) Reference signal, (**b**) signal reflected from sample G3, (**c**) their power spectra and (**d**) resultant reflectance. Insets show the refractive index profile.

The THz reference pulse (*E*_0_(*t*)) reflected from the interface constituted by the upper surface of the window and air (Figure 5a) lasted about 0.4 ps (full-width at half-maximum) and was apodised with the Blackman–Harris 4th-order asymmetrical window.

The pulse had a positive phase, because the beam was incident from the higher refractive index area on the lower one (*n_Q_* > *n*_0_), which can be explained by the Fresnel equations. The reflected signals were calculated by averaging 30 sequentially acquired signals. Total measurement time was 1 s. The signals were 40 ps long and measured with 2048 points, although for clarity we have mostly shown the most important 10-ps-long part of the signals. *E*_0_(*t*) was next Fourier transformed and the resultant power spectrum (|*E*_0_(*ω*)|^2^) had the useful spectral range from 0.1 to 2.0 THz with signal to noise ratio of about 55 dB at 0.6 THz (Figure 5b).

## 5. Samples—Analyses of Measurement Results

This section includes measurement results and analysis of samples listed in Table 1.

### 5.1. Signal Processing

The THz signal *E*_1_(*t*) and its power spectrum (|*E*_1_(*ω*)|^2^) corresponded to the scenario when the sample touched the window (Figure 5c). For sample G3, made of the fingerprinted gelatin, the reflected pulse featured the negative phase because *n_Q_* < *n_sam_* (Figure 5b). Figure 5d presents the relative power reflectance *R* = *r*^2^ = |*E*_1_(*ω*)|^2^/|*E*_0_(*ω*)|^2^, where *r* is the relative amplitude reflection coefficient. *R* can be higher than 1 because air was used as the reference. In further analysis we focus both on time signal and reflectance to distinguish a real finger from its imitations.

### 5.2. Thick Samples Results

**Figure 6:**
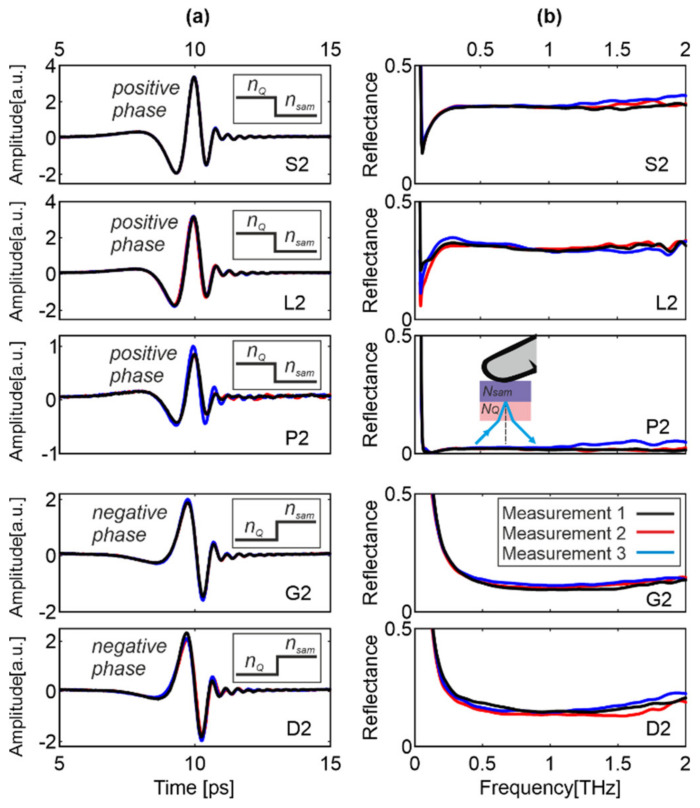
Three measurements of (**a**) reflected signal and (**b**) its reflectance for the samples: S2, L2, P2, G2 and D2 under similar conditions. Insets show the refractive index profile.

Figure 6 presents the reflected pulses and the reflectances for the rest of the thick samples, namely, S2, L2, P2, G2 and P2. Each sample was independently attached to the window three times under similar conditions. The observed fluctuations are small and result from the fact that each time a slightly different part of the sample was attached with different pressure.

For the samples from Group I, the positive phase is observed, because *n_Q_* > *n_sam_*. The reflectances decrease rapidly, reach a sharp minimum at about 0.1 THz, increase, and then reach an almost constant value. On the contrary, samples D1 and P2 (Group II) feature the negative phase (*n_Q_* < *n_sam_*), while their reflectances decrease monotonically, reach a wide minimum, and increase slightly at higher frequencies. For sample G3 (Figure 5) and G2 (Figure 6), both the reflected signals and reflectances are very similar, which proves that the fingerprint pattern does not affect the THz signal.

### 5.3. Thin Samples Results

**Figure 7:**
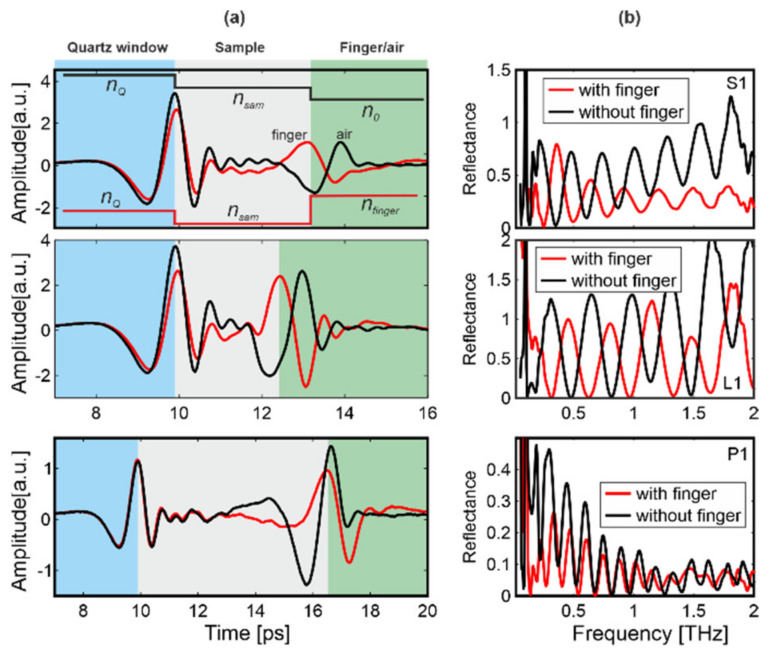
(**a**) Reflected signal and (**b**) its reflectance for the samples: S1, L1 and P1 for scenario with and without finger.

Attenuation of Play-Doh and gelatin was very high and hence the THz radiation was strongly suppressed even for the thin samples G1 and D1. The resultant characteristics were very similar to those presented for thick samples and therefore we limited further analysis only to the samples from Group I—S1, L1 and P1 (Figure 7).

In this case, a part of the propagating THz radiation was reflected back from the interface quartz/sample (first echo), which followed the same rules as for thick samples. The rest of the radiation propagated through the sample and was reflected from the back of the sample, creating a second interface (second echo). If the sample is pressed by the finger, the phase is negative (*n_sam_* < *n_finger_*). The lack of the finger (air) manifests itself as the positive phase (*n_sam_* > *n*_0_). Thus, by analyzing the phase of the second echo we can also determine if the overlay was touched by the finger or was surrounded by air. Moreover, the presence of the second echo clearly indicates a spoofing attempt. Due to the fact that the reflected signal consists of two separated pulses (first and second echoes), the reflectances consist of oscillations, which also can be used as an alarm criterion.

### 5.4. Simulations

The relative coefficient *r*, described above, can also be represented as the ratio of absolute coefficients [53]:

(1) $$r=\frac{\left\lfloor E_{1}\left(\omega \right)\right\rfloor }{\left\lfloor E_{0}\left(\omega \right)\right\rfloor }=\frac{r_{sam}}{r_{0}}$$

where:

(2) $$r_{o,sam}=\frac{{N_{Q}}^{2}cos\Theta_{1}-N_{Q}\sqrt{N_{o,sam}^{2}-N_{Q}^{2}{cos}^{2}\Theta_{1}}}{{N_{Q}}^{2}cos\Theta_{1}+N_{Q}\sqrt{N_{o,sam}^{2}-N_{Q}^{2}{cos}^{2}\Theta_{1}}}$$

*r_o_*_,_*_sam_* represents the absolute amplitude reflection coefficients for interface quartz/air and quartz/sample, respectively, and originate from the Fresnel equations. *N_sam_* is the complex refractive index of the sample. Thus, knowing the reference signal *E_o_*(*t*), *N_Q_*, *N_sam_*, and using Equations (1) and (2) we can calculate the signal reflected from the sample, *E*_1_(*t*).

**Figure 8:**
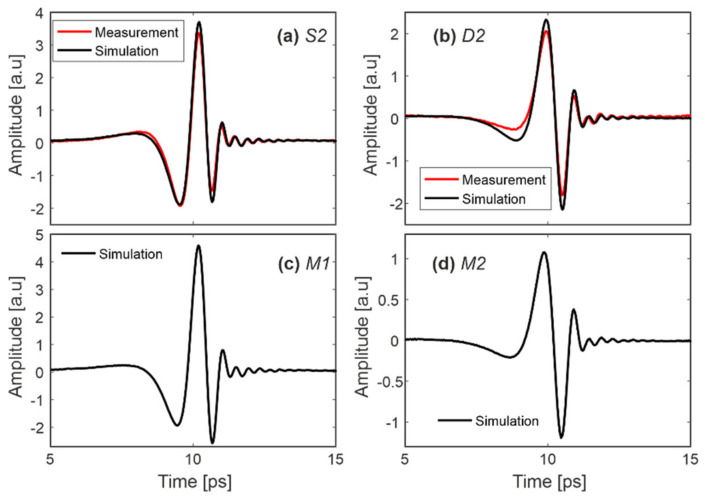
Reflected signal: measurement and simulation for (**a**) silicone and (**b**) Play-Doh, as well as simulation for (**c**) M1 and (**d**) M2 samples.

Figure 8a,b show that the measured signals reflected from the samples S2 and D2 (the same as shown in Figure 6) are very similar to simulations performed for corresponding materials with the complex refractive indices presented in Figure 3. It is hence believed that Equations (1) and (2) correctly describe the analyzed phenomenon, while small differences are probably connected with the fact that the sample is pressed to the window by a finger, which could slightly modify the complex refractive index determined under pressure-free conditions.

The idea behind this part of the research was to simulate the THz signal if someone in future uses materials with other parameters than those in Group I and Group II. Although we think that we selected the most popular set of materials for fingerprint spoofing (Table 1), it could be that imposters will use materials with other unknown today parameters. Since we have not found in the literature any significantly different spoofing materials, for further simulations we “developed” two artificial materials, M1 and M2, having both silicone and Play-Doh features according to formulas: *N*_*M*1_ = *n_Silicone_* + i/*_Play-Doh_* and *N*_*M*2_ = *n_Play-Doh_* + i/*_Silicone_*.

The simulations performed (Figure 8c,d) show that the signal reflected from sample M1 is similar to that from sample S2 but has higher amplitude. The signal corresponding to sample M2 is similar to D2 but has lower amplitude. Due to similarities to the real samples, the classification algorithm (Section 6) could be also applied to the presented artificial materials.

## 6. Finger Skin—Analyses of Measurement Results

In this section, we present analysis of experimental results for real human fingers.

### 6.1. Person 1

**Figure 9:**
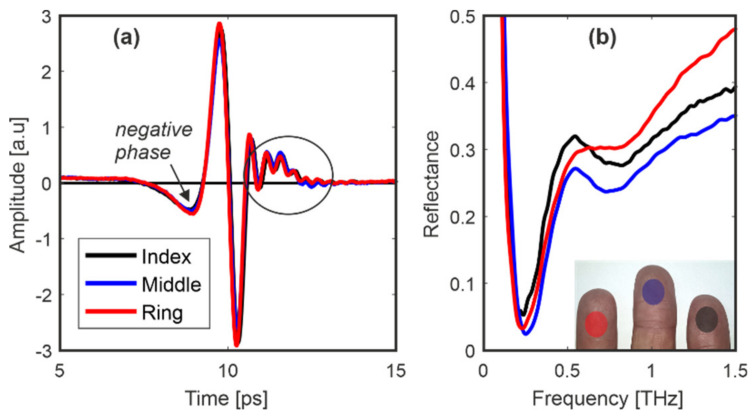
Person 1: signals reflected from (**a**) index, middle and ring fingers and (**b**) their reflectances. Inset shows the scanned fingers.

We thoroughly analyzed person 1′s fingers in different configurations and conditions. Figure 9 presents results for index, middle and ring fingers. Here, the phase of the reflected signal is also negative because *n_Q_* < *n_finger_*.

**Figure 10:**
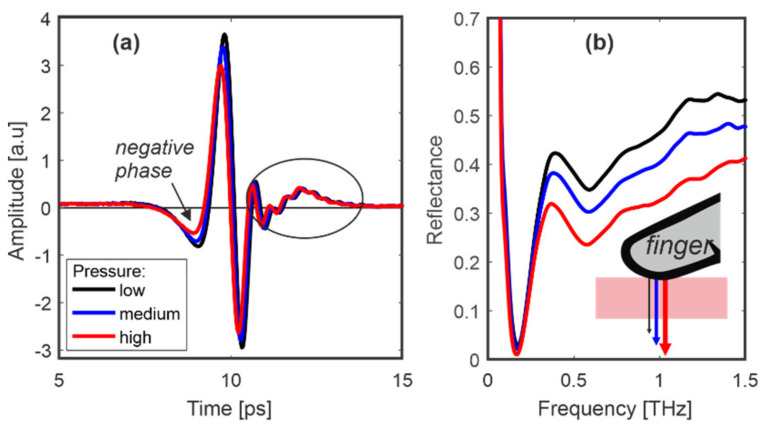
Person 1: signals reflected from (**a**) the middle finger and (**b**) their reflectances for low, medium and high pressure.

**Figure 11:**
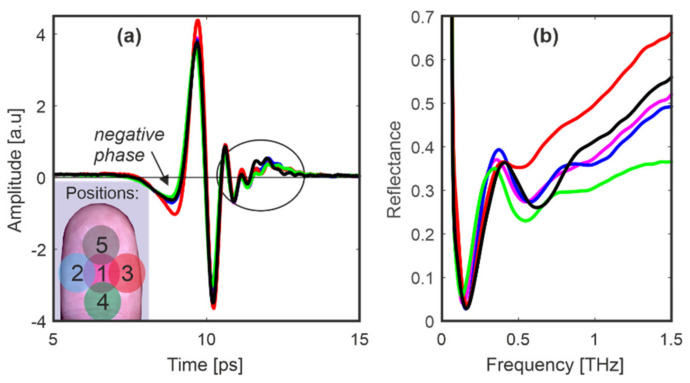
Person 1: signals reflected from (**a**) the middle finger pad and (**b**) their reflectances for five positions. Inset shows the scanned regions.

In the case of the samples (Figure 5 and Figure 6), the part of the reflected signal right after the main pulse has only small rapidly decreasing oscillations symmetrical around 0. In Figure 9, Figure 10 and Figure 11, the discussed part of the signals (marked with ellipse) is modified—about 2 ps right after the main pulse we can observe small and expanded pulse with evanescent oscillations. We think that this is a characteristic feature, which distinguishes a real finger from its imitations or overlays. The reflectance features a characteristic shape with the main minimum at about 0.25 THz and some further oscillations visible up to about 0.7 THz. The reflectance is significantly different from those presented for imitations in Figure 5 regarding the overall shape (ascending vs. flat curve for frequencies above 0.5 THz) as well as shape and position of the minimum.

**Figure 12:**
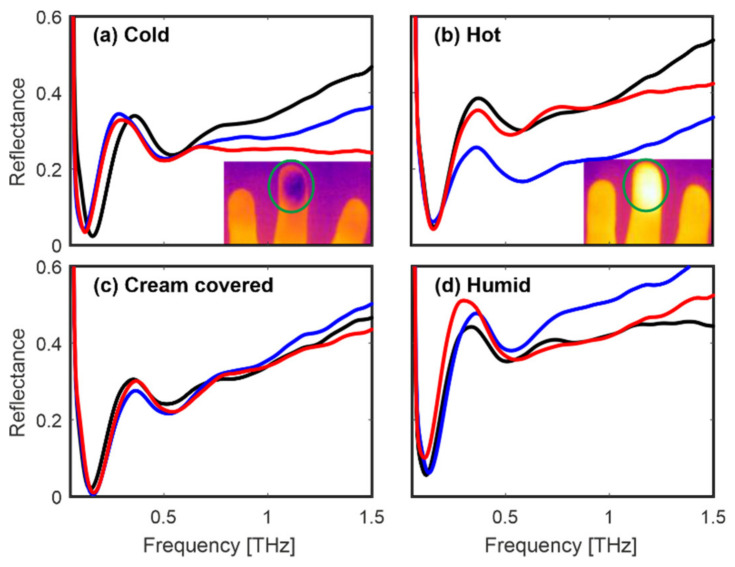
Person 1: reflectances of the middle finger for four conditions: (**a**) cold, (**b**) hot, (**c**) cream covered, and (**d**) humid. Insets show a cold and hot finger image recorded by a thermal camera.

Figure 10, Figure 11 and Figure 12 present results for the middle finger of person 1. First, the finger was placed on the window and pressed with increasing force without picking up (Figure 10). The resultant amplitude of the pulse and reflectance decreased. The same measurements were repeated for the other persons’ fingers and gave similar dependence.

In the next tests, five different parts of the finger pad (see inset in Figure 11) were scanned. It can be deduced that the whole finger pad interacts similarly with the THz pulse while some discrepancies are probably connected with the fact that persons applied different pressure (Figure 11). Finally, we also changed the conditions of the finger. It was cooled down and heated up by about 5 °C by touching a cold and hot surface (inset in Figure 12a,b respectively).

Next, the finger was covered with hand cream and we waited 5 min until it was absorbed. Moreover, the finger was immersed in water for a minute, wiped, and after one minute it was put on the quartz window, to mimic sweating. Although the described operations caused some changes in amplitudes of reflectances, the characteristic setup of minima and maxima remained constant (Figure 12c,d). Positions of the first minimum of the reflectance shifted in the range of about 0.1–0.3 THz while positions of the first maximum shifted in the range of about 0.3–0.5 THz.

To summarize, the measurements of person 1’s fingers proved that the characteristic distribution of minima and maxima changes slightly under the influence of various factors which can occur in real-life conditions.

### 6.2. Persons 2–6

**Figure 13:**
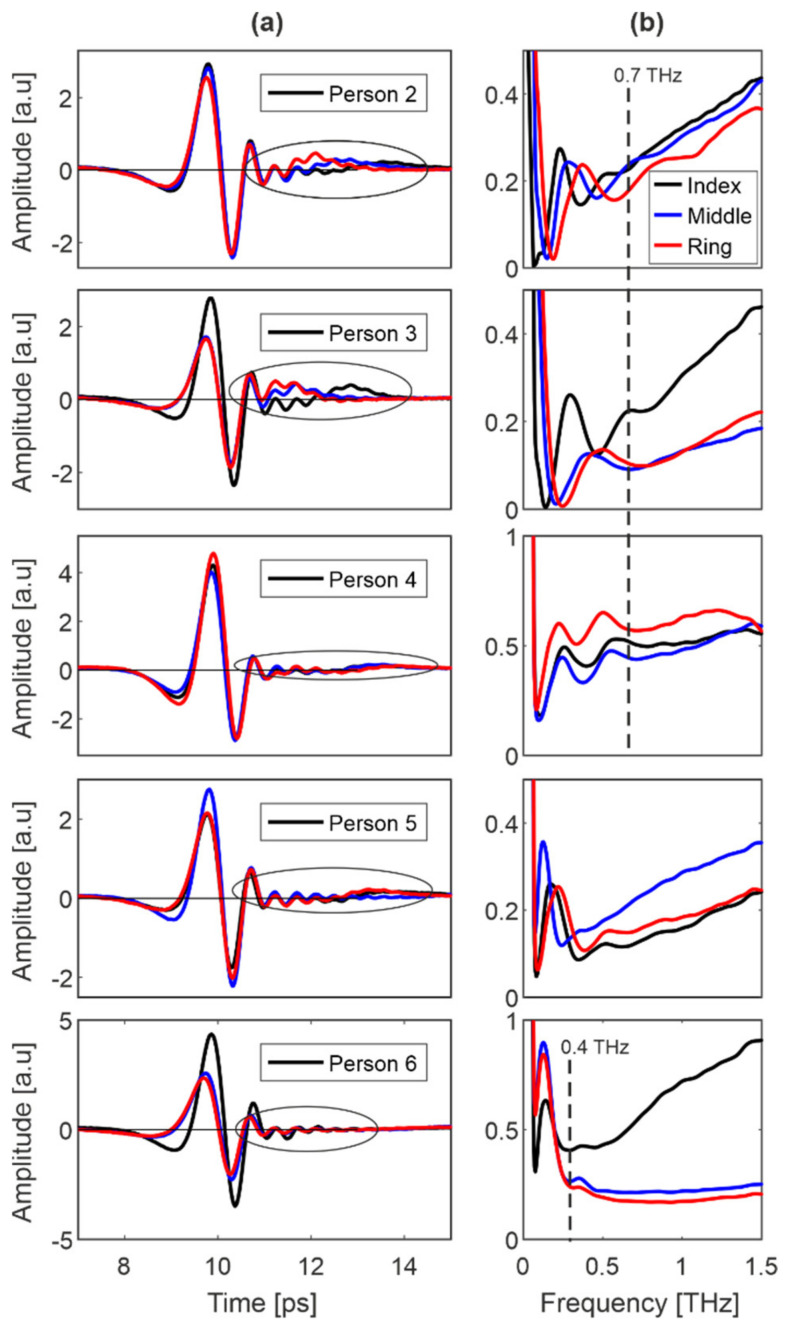
Person 2–6: signals reflected from (**a**) index, middle and ring fingers and (**b**) their reflectances.

Figure 13 presents the results for index, middle and ring finger of five representative persons named 2–6. We limited the presented data because the results for other persons were similar.

As shown previously, the modification in the reflected signal right after the main pulse (marked with the ellipse) can be noticed. Due to the diversity of the group, the variety of modifications is higher—they are bigger or smaller and above or below 0, as well as closer or farther from the main pulse. Simultaneously, each person possesses her/his own shape of reflectance with the set of minima and maxima, which stays relatively constant for the scanned finger pads. For some persons (e.g., person 6), these extrema occupy the range below 0.3 THz, and for others (e.g., persons 2–4), the range below 0.7 THz. However, in the range below 0.7 THz, the reflectance characteristics of the finger pads differ significantly from the characteristics of samples (Figure 5 and Figure 6) and therefore they are further studied as a comparative criterion.

### 6.3. Deconvolution-Based Discussion

For further signal analysis, we used the deconvolution technique, which is widely used in TDS systems operating in reflection configuration. One can deconvolve the complicated signal reflected from a multilayer sample and determine the interfaces between media by calculating the response function (*rf*) [54]:

(3) $$rf=FFT^{-1}\left(FFT\left(filter\right)\frac{FFT\left(E_{1}\right)}{FFT\left(E_{0}\right)}\right)$$

where *E*_1_(*t*) and *E*_0_(*t*) are the signals reflected from the sample and the reference, respectively (Figure 5), while a filter is used to suppress noise, usually using the double Gaussian filter described as:

(4) $$filter=\frac{1}{HF}exp\left(\frac{-t^{2}}{HF^{2}}\right)-\frac{1}{LF}exp\left(\frac{-t^{2}}{LF^{2}}\right)$$

where *LF* = 12 and *HF* = 2048 are the low- and high-frequency coefficients, respectively, selected by the trial and error method.

**Figure 14:**
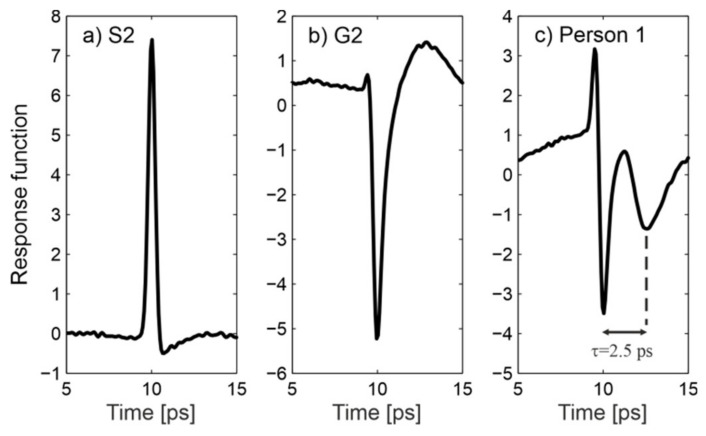
Response function for: (**a**) sample S2, (**b**) sample G2 and (**c**) person 1.

Figure 14 presents response functions calculated for samples S2 and G2 (see Figure 6 for corresponding reflected signals) and for the middle finger of person 1 (see Figure 9). Both samples feature a Gaussian-like response function. Its sign is governed by the phase of the reflected signal. For the finger pad, one can notice the additional pulse (echo) which can be attributed to reflection from an internal interface. We estimated the depth *d* ≈ 200 μm at which this interface is located using the formula *d* = *c* ∙ *τ*/2*n*cos(*θ*_1_)), where *c* is the speed of light, *τ* = 2.5 ps is the time distance between pulses, and *n* ≈ 2 is the mean refractive index of skin (Figure 3). This depth corresponds to the thickness of the stratum corneum and is in compliance with previous findings [37]. The inner layers of the epidermis have a higher water content [35,36,55], which explains their higher refractive index, creating the interface and the phases of both reflections. Moreover, sweat ducts (Figure 1) have been proven to function as helical antennae with a resonance frequency of about 0.45 THz [37]. In this range, the observed oscillations of the reflectance also take place.

To summarize, we provided a basic explanation of the observed phenomena, but we believe that further studies are required to better understand the interaction of THz radiation with the complicated structure of friction ridge skin.

## 7. Spoofing Detection Algorithm

As a result of analysis presented in Section 5 and Section 6, we propose two spoofing detection methods. The first method uses several feature points extracted from time and frequency signals to distinguish spoof fingerprints from genuine samples. The second detection method uses frequency domain signals to feed the deep learning classification model, as well as time domain data for a secondary check. Since no datasets of fingerprint spoofs in the THz range are publicly available, we collected an in-house dataset of spoofs and live fingerprints using the THz TDS setup. In total, we acquired 280 measurements, including 144 measurements of genuine fingers (persons 1–16, three fingers measured three times each) and 136 measurements of spoofed samples (materials listed in Table 1 with various thicknesses measured in different ways). As a result of the time–frequency analysis, the spoofing samples were divided into two groups according to their properties. The first group of spoof materials included silicone, latex and plasticine, while the second group of materials contained gelatin, Play-Doh and water-based samples. Algorithms were evaluated with regards to true detection rate (TDR) and false detection rate (FDR).

### 7.1. Time–Frequency Features Method

The first spoofing detection method is a combination of two criteria based on the time and frequency domain signals. The time and frequency domain analysis provided information on possible separation between live and spoof samples. We developed a two-criteria algorithm with predefined time–frequency features.

**Figure 15:**
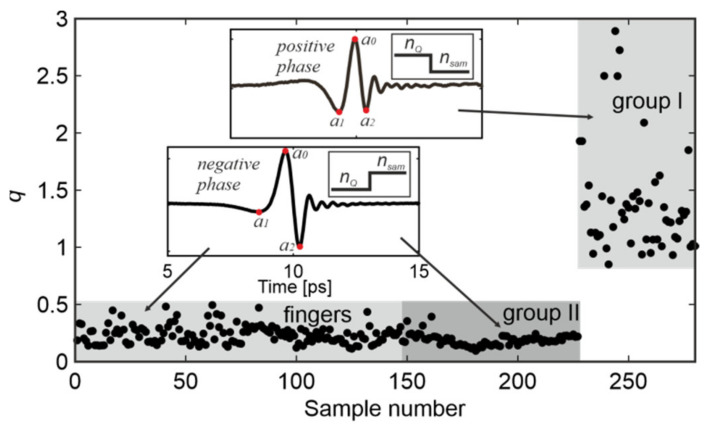
The *q*-coefficient calculated for 280 time signals. Insets show determination of coefficient *q* Figure 2. Fingers (negative phase), respectively.

First, the algorithm analyzes the time domain signals to determine the ratio between two local minima of the signal. The algorithm determines the signal phase by finding the maximum (*t*_0_, *a*_0_) of the reflected signal in the fixed range of about 2 ps (insets in Figure 15). This range is determined by the position of the upper surface of the quartz window and is equal to 9–11 ps in the case considered. Next, the algorithm looks for two minima lying on both sides of the maximum (*a*_1_ and *a*_2_) in the vicinity of *t*_0_ (±2 ps). Finally, the coefficient *q* = *a*_1_/*a*_2_ is calculated (Figure 15).

We can note that for Group I (positive phase), *q* > 0.85 with a mean value *q_G_*_1_ = 1.38 ± 0.46. For the remaining measurements with the negative phase, *q* < 0.5 with a mean value of *q_G_*_2_ = 0.19 ± 0.05 and *q_F_* = 0.24 ± 0.08 for the Group II samples and fingers, respectively. For genuine fingers, the variability of results is higher due to the higher randomness of finger placement. Finally, the *q*-coefficient (*q* > 0.85) analysis is considered as the first (time domain) criterion.

**Figure 16:**
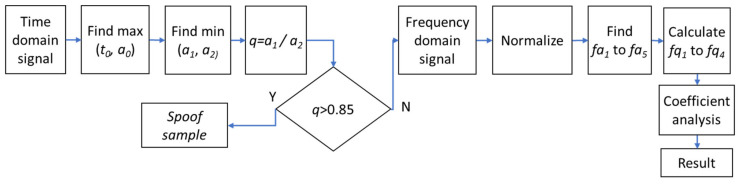
Block diagram of time–frequency domain coefficient features algorithm.

Since Group II is not separable from the genuine fingers based on time domain signals, all samples that do not pass the first criterion (*q*) are subject to the frequency domain analysis, which is based on a five feature points scheme. The block diagram of the time–frequency features algorithm is presented in Figure 16.

**Figure 17:**
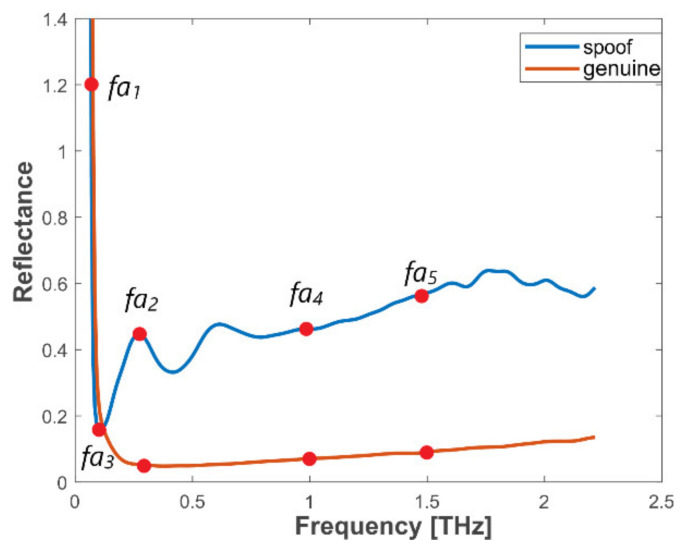
Reflectance with feature points for genuine and spoofed samples.

Analysis of frequency domain reflectance graphs of different spoof materials and genuine fingerprints allowed us to select five points which well indicate the differences between frequency signals of spoofs and genuine samples. Based on these five feature points, we developed a method for sample separation which is the second criterion of the proposed algorithm. The points extracted from normalized measurement data include *fa*_1_ to *fa*_5_, where *fa*_1_ is the maximum amplitude in the range 180–500 GHz, *fa*_2_ is an amplitude at frequency of 300 GHz, *fa*_3_ is a minimum amplitude in the range 180–500 GHz, *fa*_4_ is an amplitude at frequency of 1000 GHz, and *fa*_5_ is an amplitude at frequency of 1500 GHz. Based on values *fa*_1_–*fa*_5_, coefficients *fq*_1_ = *fa*_1_/*fa*_2_, *fq*_2_ = *fa*_1_/*fa*_3_, *fq*_3_ = *fa*_2_/*fa*_3_ and *fq*_4_ = *fa*_5_/*fa*_4_ are calculated. We determined that the following coefficient values should be taken to distinguish Group II samples from genuine fingerprints: *fq*_1_ > 4.5, *fq*_2_ > 3.5, *fq*_3_ < 1.21 and *fq*_4_ < 1.1. The second (frequency domain) criterion is fulfilled when all four coefficients pass. Localization of feature points is presented in Figure 17.

The samples from Group I constitute 40% of all the samples. The first criterion (*q*) based on the time domain analysis provides a TDR of 100% for spoof materials of Group I. This implies that only genuine samples and Group II samples are subject to the second criterion analysis. The second criterion analysis (*fq*_1_–*fq*_4_) based on frequency domain signals applied to remaining samples provides a TDR of 86.9% for Group II (60% of all samples with gelatin, Play-Doh, the water-based samples) and a TDR of 97.9% for genuine samples. The cumulative result of all samples after the two-criteria analysis achieved a TDR of 87.9% at FDR = 3.9%. All the results achieved by the first spoofing detection method are presented in Table 2 with respect to each group of materials.

The key finding of the results presented above is that spoof materials of Group II are of much higher variability with regards to the reflectance. The method based on two-criteria analysis is able to provide a high total TDR of almost 88% without applying a machine learning scheme.

### 7.2. Deep Learning-Based Method

The second version of the spoofing detection method uses two criteria, which are based on the deep learning classification and the time domain analysis. The deep learning classifier analyzes frequency domain signals. The second criterion uses the *q*-coefficient extracted from time domain signals. The neural network (NN) classifier was trained and tested on a relatively small dataset; however, it aims to give a proof of concept.

The deep learning classifier used during this study is based on the ResNet-18 network [56]. The ResNet-18 is a compact residual neural network which uses identity shortcut connections to jump over some layers. The ResNet networks have been developed as a solution for vanishing gradients [56]. Since the collected dataset of samples is relatively small, we have chosen this network as it has been proven to be less prone to overfitting.

The network analyzes the frequency graphs in order to classify them as spoofs or genuine. The algorithm aims to provide a good separation rate and high generalization across unknown samples. During training of the classifier, a simple transfer learning scheme was applied. The network model was first trained on the ImageNet dataset in order to provide general representation of various objects and retrained on a variety of graphs presenting genuine as well as spoofed samples. Normalized graphs of signals in the frequency domain were used as an input to the deep learning model. The normalization was performed both in amplitude (reflectance) and frequency domains.

First, the deep learning method determines whether the frequency domain graph belongs to a genuine or spoofed sample. Next, for all genuine results, the algorithm checks the *q*-coefficient extracted from the time domain data. If the *q*-value is higher than 0.85, the sample is likely to belong to Group II and therefore the result is reclassified as spoofed.

**Figure 18:**
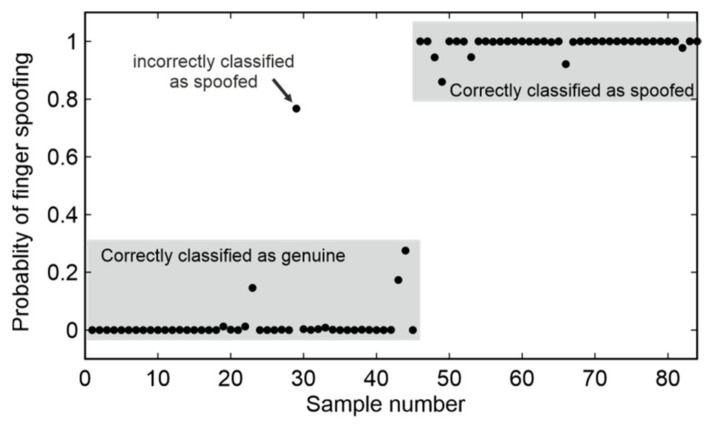
Results of the five-fold cross validation.

Two training and validation approaches were applied. In the first approach, the five-fold cross-validation method was applied. A random selection of 70% of the measurements was used as the training set (196). The remaining 30% constituted the testing set (84). After training, the NN classified the sample as genuine/spoofed and determined the probability of spoofing in the range 0–1. As a result of the NN classification, no samples were falsely accepted and 2.4% were falsely rejected. After analysis of the second criterion (*q*-coefficient) applied to the rejected samples, the final TDR = 98.8% and FDR = 1.2%. The mean results of the five-fold cross-validation are presented in Figure 18 and Table 3.

As a second validation approach, the cross-material validation was applied to verify the generalization of the proposed method. During the cross-material validation, the training and testing split was based on known and unknown materials. In all the cross-material scenarios, graphs of reflectance in frequency domain of one group of materials were partitioned to the classifier for training, and the spoof impressions of the second group of “unknown” materials were kept aside for testing. In this manner the generalization capability of the spoofing detection method was thoroughly assessed. Since there are two groups of different spoof materials, we conducted two different cross-material validations using ResNet-18 with and without the *q*-coefficient criterion. Collective results of the five-fold cross-validation and the cross-material validation are presented in Table 3.

The results provided above indicate that the method achieves high performance when trained on all known materials. However, since the method is based on two-criteria analysis, we have made calculations for both the deep learning classifier, as well as for deep learning classifier with *q*-coefficient analysis as the second criterion.

The cross-material experiments on the deep learning model revealed that spoof materials of Group I also generalize very well to materials of Group II, achieving over 93% TDR. The reverse scenario shows that performance of a classifier trained on Group II of spoofs decreased significantly when tested on samples from Group I. As a second round, we also conducted experiments with classifier and *q*-coefficient analysis. The time domain analysis improved the overall performance of the proposed method for spoofs of Group I, while the results for Group II remained unchanged.

The presented spoofing detection method is not only software based but relies on analysis of reflectance signals registered with terahertz time–domain spectrometer. Our method does not analyze the fingerprint images to detect spoofs but uses specific properties of terahertz radiation in time–domain setup to detect additional layers attached to the fingerprint.

We have compared performance of presented method with results provided during last two editions of LivDet. In LivDet 2017, the best performing algorithm achieved overall accuracy of 95.25% with over 93% of accuracy during cross-material validation for each of the test sets. The best performing liveness detection algorithm investigated during LivDet 2019 achieved overall score of 96.17%. However, LivDet 2019 was focused to investigate how the integration of a liveness detection system with a fingerprint authentication system may impact the performance.

Our study concerns spoofing detection without integration with fingerprint reader. The first method based on time–frequency features analysis achieved TDR of 87.9% and FDR = 3.9%. The second method, based on the deep learning classifier applied to reflectance spectra, with a second criterion based on reflected signals in the time domain achieved TDR of 98.8%.

## 8. Conclusions

In this study, using the time domain spectroscopy setup in the reflection configuration, we studied the interaction of terahertz radiation with the friction ridge skin of finger pads and with spoofed samples. We prepared homemade imitations using five commonly used spoof materials and determined their complex refractive indices, which were successfully used in further calculations. We proved that both the reflected time signals and the reflectance spectra of the imitations differ significantly from the living fingers of 16 persons. The deconvolution-based analysis provided the basic explanation of the observed phenomena—unique back reflection from the inner layer of friction ridge skin. We also studied the influence of finger condition on its time signal and reflectance spectrum. It was proved that different downforce, place, temperature, and humidity had little impact on the measured characteristics. We collected fingerprints of 16 persons who differed in age and gender and samples of five popular spoofing materials of different thickness. Both samples and fingers were attached to the scanning window in different ways.

The time and frequency domain analyses provided good separation between spoofs and live samples. Based on the conducted analysis, we proposed two methods for spoof detection. The first method was based on time–frequency features analysis. This method is easy to interpret and achieved a TDR of 87.9% and FDR = 3.9%. The second method was based on the deep learning classifier applied to reflectance spectra, with a second criterion based on reflected signals in the time domain. The second method with five-fold validation provided excellent classification with a TDR of 98.8%. The second method was also validated using the cross-material scenario, achieving slightly lower results of TDR = 98.7% and TDR = 93.2% for silicone, latex and plasticine samples (Group I) and gelatin, Play-Doh and water-based samples (Group II), respectively.

Our results clearly proved that terahertz technology can be successfully applied for high accuracy hardware-based fingerprint spoofing detection. All the experiments were carried out using rather bulky scientific laboratory equipment. The spectrometer itself is not compact, but the measurement is fast (below one second) and easy. In addition, the developed system can be easily integrated with the existing optical fingerprint readers, which camera can be installed in the free space between the lenses (see Figure 4). However, the possibility to reduce the size and the price of the measurement setup is the key issue for real world application. The authors are aware that the study was conducted for a limited number of persons and samples. Further research should focus on expanding the measurement population. On one hand, this will provide a more varied training set for the deep learning method and, on the other, will show the usefulness, but also the limitations, of the proposed terahertz finger spoofing detection technique.

## Figures and Tables

**Table 1:** Lists of samples.

Name	Abbr.	Material/Manufacturer	Thickness [mm]
**Silicone1**	S1	Silicone mask cover; Litfly, China	0.5
**Silicone2**	S2	Congealed sanitary silicone; Den Braven, UK	3
**Latex1**	L1	Latex glove	0.5
**Latex2**	L2	Congealed liquid latex; Smiffys, UK	3
**Plasticine1**	P1	Ordinary plasticine from a stationery store	0.5
**Plasticine2**	P2		3
**Gelatin1**	G1	Homemade congealed mixture of gelatin, water and glycerin	1
**Gelatin2**	G2		3
**Gelatin3**	G3	As above, fingerprinted	2–3
**Play-Doh1**	D1	Mixture of flour, water, salt, borax and mineral oil; Hasbro, USA	0.5
**Play-Doh2**	D2		3

**Table 2:** Results of the time–frequency feature algorithm.

Samples	Group I (%) ^1^	Group II (%) ^2^	Genuine (%) ^2^	Total (%) ^3^
TDR	100.0	86.9	97.9	87.9
FDR	0.0	10.5	2.1	3.9

^1^ results based on the first (time domain) *q*-coefficient criterion; ^2^ results based on two-criteria analysis; ^3^ cumulative results of two-criteria analysis.

**Table 3:** Validation results of the deep-learning algorithm.

Validation Method Samples	Five-Fold Cross-Validation	Cross-Material Validation			
		ResNet-18		ResNet-18 and *q*-Coefficient	
	All (%)	Group I (%)	Group II (%)	Group I (%)	Group II (%)
TDR	98.8 ± 1.2	55.8	93.2	98.7	93.2
FDR	1.2 ± 1.2	1.2	3.9	1.3	3.9
